# No risk reduction for *Plasmodium vivax* malaria in sickle cell disease

**DOI:** 10.1002/ccr3.1507

**Published:** 2018-04-14

**Authors:** Bushra Moiz, Ayesha Majeed

**Affiliations:** ^1^ Section of Hematology and Transfusion Medicine, Pathology and Laboratory Medicine The Aga Khan University Karachi Pakistan

**Keywords:** Hemoglobinopathy, malaria, sickle cell disease, vivax malaria

## Abstract

Hemoglobin S is known to protect against uncomplicated *Plasmodium falciparum* malaria. However, there is paucity of the literature regarding interaction of HbS and other malaria species. Usually, *P. vivax* malaria is a relapsing condition, and without radical cure with primaquine, recurrence may be observed even with hemoglobin S.

A 26‐year‐old man and known patient of sickle cell disease presented with high‐grade fever, chills, and bone pains since few days in 2016. Physical findings included marked pallor, hepatosplenomegaly, and short right middle finger possibly representing childhood dactylitis (Fig. 1 panel A). His transfusion was initiated at the age of one and a half years and since then received occasional red cells support. He was treated with hydroxyurea for past few years. Past history was significant for recurrent vivax malaria in 2010 and 2011 and was treated with artemether. He did not receive any primaquine therapy for radical cure. Current complete blood counts showed hemoglobin 5.4 g/dL, Hct 16.1, MCV 62.2 fl, MCH 20.8 pG, white cells 16.5 × 10^9^/L, neutrophils 47%, lymphocytes 41%, monocytes 10%, eosinophil 2%, and platelets 203 × 10^9^/L. Serum ferritin was 2848.5 ng/mL, serum calcium 9.4 mg/dL, serum phosphate 3.09 mg/dL, alanine transaminase 60 U/L, and creatinine 0.5 mg/dL. Peripheral blood film displayed (Fig. 1 panel B) hypochromic microcytic red blood cells, anisocytosis, poikilocytosis, occasional sickle cells, and features of hyposplenism (Howell–Jolly bodies, nucleated red blood cells, and target cells). The smear also showed various stages of *Plasmodium vivax* (Fig. 1 panel C: gametocyte; single arrow and D: schizont; single arrow and trophozoite; double arrows). High‐performance liquid chromatography showed Hb S: 90.5%, Hb F: 4.4%, and Hb A2: 5.1% consistent with sickle/β‐thalassemia. DEXA scan showed normal *T* and *Z* score. He was nonreactive to anti‐HIV, HBsAg, and anti‐HCV antibodies. He was treated successfully with artemisinin, and primaquine was administered to prevent recurrence. The patient was afebrile at his last follow‐up in 2017 with a negative ICT malaria test.

**Figure 1:**
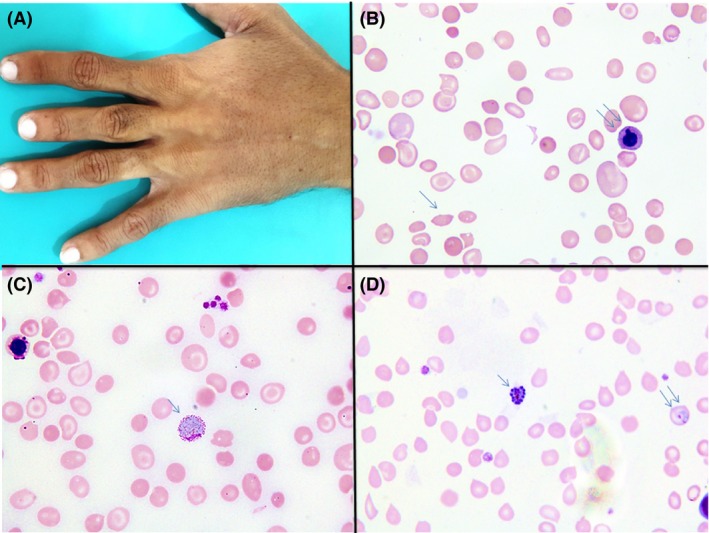
Panel A shows short right middle finger possibly representing childhood dactylitis. Panel B shows peripheral blood film displaying hypochromic microcytic red blood cells, anisocytosis, poikilocytosis, occasional sickle cells, and features of hyposplenism (Howell–Jolly bodies, nucleated red blood cells, and target cells. Panel C shows gametocyte of *Plasmodium vivax*. Panel D shows schizont (single arrow) and trophozoite (double arrows) of *P. vivax*.

Hemoglobin S (HbS) reportedly confers protection against uncomplicated *P. falciparum* malaria in sickle cell trait 1. Although exact defensive mechanisms are not clear, a number of biochemical and immunological mechanisms have been proposed 2. Spleen also plays a mechanical role in phagocytosis of parasitized red blood cells 3. In contrast to *P. falciparum* malaria, no studies investigated the protective effect of Hb S against *P. vivax* malaria 4. Latter is by its very nature a relapsing infection unless treated by hypnozoicidal agents 5.

We believe that, in our patient, Hb S did not confer any protective role, and primary reason for recurrent *P. vivax* malaria was failure to treat dormant stages or hypnozoites. Hyposplenism might have contributed to malaria relapse to some extent. Moreover, given the preference of this parasite for reticulocytes, it does not seem particularly surprising that this patient may have contracted the condition. More research is needed to understand the interaction between HbS and other malaria species.

## Authorship

BM: diagnosed the case and wrote manuscript. AM: took photographs and wrote part of manuscript.

## Conflict of Interest

The authors declare that they have no conflict of interest.
